# Patients’ Trust in Health Systems to Use Artificial Intelligence

**DOI:** 10.1001/jamanetworkopen.2024.60628

**Published:** 2025-02-14

**Authors:** Paige Nong, Jodyn Platt

**Affiliations:** 1 Division of Health Policy and Management, University of Minnesota School of Public Health, Minneapolis; 2 Department of Learning Health Sciences, University of Michigan Medical School, Ann Arbor

## Abstract

This survey study evaluates whether US adults trust health systems to use artificial intelligence responsibly and examines characteristics associated with attitudes related to the use of artificial intelligence in health care.

## Introduction

The growth and development of artificial intelligence (AI) in health care^1,2^ introduces a new set of questions about patient engagement and whether patients trust systems to use AI responsibly and safely. The answer to this question is embedded in patients’ experiences seeking care and trust in health systems.^3^ Meanwhile, the adoption of AI technology outpaces efforts to analyze patient perspectives, which are critical to designing trustworthy AI systems and ensuring patient-centered care.^4,5^

We conducted a national survey of US adults to understand whether they trust their health systems to use AI responsibly and protect them from AI harms. We also examined variables that may be associated with these attitudes, including knowledge of AI, trust, and experiences of discrimination in health care.^6^

## Methods

This cross-sectional analysis used data from an original survey fielded with a nationally representative sample of US adults via the National Opinion Research Center’s (NORC’s) probability-based AmeriSpeak Panel from June to July 2023. Poststratification survey weights using the Current Population Survey were applied to produce national estimates. Pretesting and cognitive interviews were conducted to ensure content validity and accessibility. Reporting followed the STROBE guideline. NORC obtained participants’ written informed consent, and the study was determined to be unregulated by the University of Michigan IRB.

Primary outcomes were responses to survey questions asking whether patients trust their health care system to (1) use AI responsibly and (2) ensure that an AI tool would not harm them, both measured on a 4-point Likert scale (1 = not true, 4 = very true). These questions were used to create binary measures equal to 1 when respondents indicated agreement (responses of very true or fairly true). We conducted multivariable logistic regressions to assess the associations between trusting systems to use AI and AI knowledge (true/false: AI tools can be programmed to make treatment recommendations to your doctor), health literacy, system trust, and previous experiences of discrimination in health care (eMethods in Supplement 1).

## Results

Among 2039 respondents, 1074 (51.2%) were female and 965 (48.8%) male; 53 (4.9%) were Asian, 540 (12.1%) Black, 519 (17.4%) Hispanic, 876 (63.1%) White, and 51 (2.4%) multiracial or other. The mean (SD) age was 48.4 (17.3) years. General trust in the health care system, on a scale of 0 to 12 with 12 indicating highest trust, had a mean (SD) score of 5.38 (2.18) (Figure). Most respondents reported low trust in their health care system to use AI responsibly (65.8%) and low trust that their health care system would make sure an AI tool would not harm them (57.7%).

**Figure.**
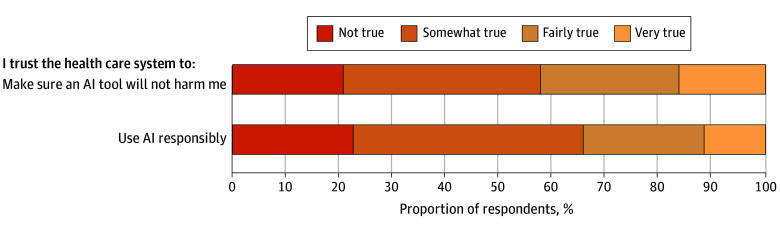
Trust in Health Care Systems to Use Artificial Intelligence (AI) Responsibly

In multivariable logistic regressions (Table), respondents with higher trust were more likely to believe that their health care system would protect them from AI harm (odds ratio [OR], 3.97; 95% CI, 3.06-5.16) and use AI responsibly (OR, 4.29; 95% CI, 3.25-5.67). Female respondents were less likely than male respondents to trust their system to use AI responsibly, but there was no difference by sex in respondents’ trust that systems would protect them from AI-related harms. Experiences of discrimination while seeking care were negatively associated with trust in systems using AI responsibly (OR, 0.66; 95% CI, 0.48-0.92) and protecting patients from harm (OR, 0.57; 95% CI, 0.40-0.81). There was no association between health literacy or AI knowledge and trust in health care systems using AI.

**Table.** Weighted Multivariable Logistic Regression Model of Trust in Systems to Use Artificial Intelligence (AI)

Variable	Trust system to use AI responsibly		Trust system to make sure an AI tool will not harm me	
	OR (95% CI)	*P* value^a^	OR (95% CI)	*P* value^a^
System trust				
Low	1 [Reference]	NA	1 [Reference]	NA
High	4.29 (3.25-5.67)	<.001	3.97 (3.06-5.16)	<.001
Experienced discrimination while seeking care				
No	1 [Reference]	NA	1 [Reference]	NA
Yes	0.66 (0.48-0.92)	.01	0.57 (0.40-0.81)	.002
AI knowledge				
Incorrect	1 [Reference]	NA	1 [Reference]	NA
Correct	1.25 (0.91-1.7)	.16	1.19 (0.92-1.54)	.18
Need health literacy support				
Always or often	1 [Reference]	NA	1 [Reference]	NA
Sometimes	0.92 (0.38-2.24)	.85	0.96 (0.40-2.32)	.93
Never or rarely	0.60 (0.26-1.43)	.25	0.87 (0.42-1.83)	.72
Sex				
Male	1 [Reference]	NA	1 [Reference]	NA
Female	0.77 (0.62-0.96)	.02	0.95 (0.75-1.20)	.67
Age, y				
18-29	1 [Reference]	NA	1 [Reference]	NA
30-44	1.26 (0.81-1.96)	.31	1.09 (0.70-1.69)	.70
45-59	0.97 (0.63-1.52)	.91	1.19 (0.77-1.84)	.45
≥60	1.36 (0.86-2.15)	.18	1.15 (0.70-1.89)	.58
Race and ethnicity^b^				
Asian, non-Hispanic	2.64 (1.46-4.78)	.001	1.46 (0.74-2.88)	.27
Black, non-Hispanic	1.25 (0.91-1.74)	.17	1.37 (1.02-1.83)	.04
Hispanic	1.28 (0.94-1.76)	.12	1.24 (0.90-1.73)	.19
White, non-Hispanic	1 [Reference]	NA	1 [Reference]	NA
Multiracial and other identities^c^	1.26 (0.55-2.91)	.58	1.04 (0.45-2.40)	.93
Education				
Less than high school	1 [Reference]	NA	1 [Reference]	NA
High school	1.23 (0.68-2.16)	.51	1.72 (1.01-2.94)	.05
Some college	1.05 (0.61-1.78)	.87	1.56 (0.87-2.79)	.13
Bachelor’s degree	0.95 (0.51-1.77)	.88	1.45 (0.79-2.66)	.23
Postgraduate study or degree	1.15 (0.63-2.11)	.65	1.69 (0.91-3.13)	.10
Annual household income				
≤$75 000	1 [Reference]	NA	1 [Reference]	NA
>$75 000	0.71 (0.54-0.94)	.02	0.92 (0.67-1.26)	.61
Health insurance coverage				
No	1 [Reference]	NA	1 [Reference]	NA
Yes	1.16 (0.67-2.00)	.60	1.05 (0.69-1.61)	.81

Abbreviations: NA, not applicable; OR, odds ratio.

^a^ Two-tailed test with significance cutoff of *P* < .05.

^b^ Race and ethnicity were self-reported by respondents; the categories available to respondents are listed in the table. Race and ethnicity data were collected to ensure sufficient representation and identify any systematic demographic differences in perspectives on AI use in health care.

^c^ Multiracial and other were options provided to respondents on the survey instrument. These categories were combined due to small sample sizes and do not represent collapsed or combined categories.

## Discussion

This analysis found low trust in health care systems to use AI responsibly and protect patients from AI-related harms. General trust in the health care system, but not health literacy or AI knowledge, was associated with these perceptions. This analysis is limited by its observational, cross-sectional design. Future work should examine this trust longitudinally and include additional validated measures of factors such as patient comfort, familiarity, and experience with AI that could be associated with the outcome. Low trust in health care systems to use AI indicates a need for improved communication and investments in organizational trustworthiness.

## Notes

Supplement 1.
eMethods.

Supplement 2.
Data Sharing Statement
