# Supplementary material for: Patients’ Trust in Health Systems to Use Artificial Intelligence
Source: JAMA Netw Open. 2025 Feb 14;8(2):e2460628. doi: 10.1001/jamanetworkopen.2024.60628 (PMC11829222; doi:10.1001/jamanetworkopen.2024.60628)
Supplement: Supplement 1. — eMethods. [file jamanetwopen-e2460628-s001.pdf]

Supplemental Online Content

Nong P, Platt J. Patients’ trust in health systems to use artificial intelligence. *JAMA Netw Open*. 2025;8(2):e2460628. doi:10.1001/jamanetworkopen.2024.60628

eMethods.

This supplemental material has been provided by the authors to give readers additional information about their work.

© 2025 Nong P et al. JAMA Network Open.

eMethods

Survey instrument
The survey included comprehension questions AI, brief scenarios, and measures of health literacy to ensure respondent comprehension. Respondents viewed a 90-second explanatory video describing what AI is and how it can be applied in healthcare. This method minimizes respondent cognitive load by replacing long text descriptions of advanced technologies and has been used by the research team for multiple national surveys. (See Nong P, Adler-Milstein J, Kardia S, Platt J. Public perspectives on the use of different data types for prediction in healthcare. J Am Med Inform Assoc. 2024 Feb 1;ocae009, Nong P, Adler-Milstein J, Platt J. How Patients Distinguish Between Clinical and Administrative Predictive Models in Health Care. 2024 Jan 10;30:31–7, Platt J, Raj M, Kardia SLR. The public's trust and information brokers in health care, public health and research. J Health Organ Manag. 2019 Nov 7;33(7/8):929–48).) The video and survey questions were tested through focus groups and cognitive interviews to ensure accessibility and content validity. The instrument was piloted with a sample from the NORC AmeriSpeak Panel (n=116) prior to fielding.

Survey measures
The measure of health literacy was adapted from previous national surveys and validation studies ("How often do you need to have someone help you when you read instructions, pamphlets, or other written material from your doctor or pharmacy?"). See Chew, Lisa D., Joan M. Griffin, Melissa R. Partin, Siamak Noorbaloochi, Joseph P. Grill, Annamay Snyder, Katharine A. Bradley, Sean M. Nugent, Alisha D. Baines, and Michelle VanRyn. 2008. "Validation of Screening Questions for Limited Health Literacy in a Large VA Outpatient Population." *Journal of General Internal Medicine* 23(5):561–66. doi: 10.1007/s11606-008-0520-5.

AI knowledge was measured by responses to a true or false question about AI use in healthcare ("True or false: AI tools can be programmed to make treatment recommendations to your doctor").

Composite measure of system trust
Trust in the healthcare system was calculated as an index measure from 13 individual survey questions spanning four key dimensions of trust; fidelity, integrity, global trust, and competence. These dimensions have been established as important aspects of trust in the literature (Taylor, Lauren A., Paige Nong, and Jodyn Platt. 2023. "Fifty Years of Trust Research in Health Care: A Synthetic Review." *The Milbank Quarterly* 1–53. doi: 10.1111/1468-0009.12598.).

The Cronbach's alpha indicated high internal consistency within each of the four dimensions of trust, indicating that index construction was appropriate. The average trust score for each respondent was calculated and summed across all four dimensions to produce the overall system trust index (see Platt, Jodyn E., Peter D. Jacobson, and Sharon L. R. Kardia. 2018. "Public Trust in Health Information Sharing: A Measure of System Trust." *Health Services Research* 53(2):824–45. doi: 10.1111/1475-6773.12654.).

We examined the correlations between each of our outcome variables (trust system to use AI responsibly, trust system to prevent harm) and the composite system trust variable. These correlation coefficients are relatively low at 0.324 and 0.318 respectively. We revised the factor analysis (with an oblique promax rotation) including all the survey items comprising the existing system trust index with the two outcome variables added. We did this to identify whether our outcome variables represented dimensions of our composite system trust measure. We found that the outcome variables did not load with any of the existing system trust measures at a factor loading of 0.4 or above, which would be required for their inclusion in the index with an existing factor. This is another indication that our outcome variables are not reflective of dimensions of the system trust index.

© 2025 Nong P et al. JAMA Network Open.
