# Supplementary material for: Patients’ Trust in Health Systems to Use Artificial Intelligence
Source: JAMA Netw Open. 2025 Feb 14;8(2):e2460628. doi: 10.1001/jamanetworkopen.2024.60628 (PMC11829222; doi:10.1001/jamanetworkopen.2024.60628)
Supplement: Supplement 2. — Data Sharing Statement [file jamanetwopen-e2460628-s002.pdf]

Data Sharing Statement

Nong. Patients' Trust in Health Systems to Use Artificial Intelligence. *JAMA Netw Open*. Published February 19, 2025. doi:10.1001/jamanetworkopen.2024.60628

Data
Data available: Yes
Data types: Deidentified participant data
How to access data: Data available for research upon request
When available: With publication

Supporting Documents
Document types: None

Additional Information
Who can access the data: Researchers whose proposed use has been approved
Types of analyses: Research
Mechanisms of data availability: After approval and with a signed data access agreement
